# Zinc for Infection Prevention in Sickle Cell Anemia (ZIPS): study protocol for a randomized placebo-controlled trial in Ugandan children with sickle cell anemia

**DOI:** 10.1186/s13063-019-3569-z

**Published:** 2019-07-26

**Authors:** Dibyadyuti Datta, Ruth Namazzi, Andrea L. Conroy, Sarah E. Cusick, Heather A. Hume, Abner Tagoola, Russell E. Ware, Robert O. Opoka, Chandy C. John

**Affiliations:** 1 Ryan White Center for Pediatric Infectious Disease and Global Health, Department of Pediatrics, Indiana University School of Medicine, 1044 W. Walnut St, R4 402D, Indianapolis, IN 46202 USA; 2 Department of Paediatrics and Child Health, Makerere University, Kampala, Uganda; 3 Department of Pediatrics, University of Minnesota School of Medicine, Minneapolis, MN USA; 4 University of Montreal, Montreal, Canada; 5 Jinja Regional Referral Hospital, Jinja, Uganda; 6 Cincinnati Children’s Hospital, Cincinnati, Ohio USA

**Keywords:** Sickle cell anemia, Zinc, Uganda

## Abstract

**Background:**

Sickle cell anemia (SCA) is the most common inherited hemoglobinopathy worldwide. Infection is a major cause of illness and death in children with SCA, especially in sub-Saharan Africa where an estimated 50–90% of affected children die before their fifth birthday. Interventions to reduce the incidence and severity of infections are needed urgently. A high proportion of adults and children with SCA are zinc-deficient, and zinc deficiency leads to impaired immunity and an increased risk of infection. Zinc supplementation has been shown to decrease the risk of infection in adolescents and adults, but there are no data on the effectiveness of zinc for prevention of infection in children < 5 years of age with SCA.

**Methods/design:**

The study will be a randomized, placebo-controlled, double-blind clinical trial in which 250 Ugandan children 1.00–4.99 years of age with SCA will receive daily zinc supplementation (10 mg oral dispersible tablet) or identical placebo for 12 months.

**Discussion:**

If this trial shows a reduction in severe or invasive infection incidence, it would be the basis for a multi-site, multi-country clinical trial to assess real-world safety and efficacy of zinc in African children with SCA. Since zinc is safe, inexpensive, and easy to administer, this trial has the potential to improve the health of hundreds of thousands of African children with SCA through reduction of infection-related morbidity and mortality.

**Trial registration:**

Clinicaltrials.gov, NCT03528434. Registered on May 17, 2018

Protocol Version: 1.0. Date: Dec 11, 2017

Sponsor: Indiana University. Sponsor’s protocol identifier, 1712339562

**Electronic supplementary material:**

The online version of this article (10.1186/s13063-019-3569-z) contains supplementary material, which is available to authorized users.

## Background

Sickle cell anemia (SCA) is an inherited hemoglobinopathy characterized by chronic hemolytic anemia and vascular occlusion. Africa is disproportionally affected: more than 240,000 of the approximately 300,000 infants born with SCA annually are born in sub-Saharan Africa [1]. Generally, the prevalence of sickle cell disease is < 1% across sub-Saharan Africa, but it contributes to around 5% of all deaths in children under 5 years of age [2, 3]. In Uganda, SCA is a major public health problem with approximately 15,000 Ugandan children born with SCA annually [4], and an estimated 50–90% of children with SCA in Uganda die before the age of 2 years [2].

Infections in children with SCA are a major cause of hospitalization and mortality. The risk of infectious complications in SCA is most evident in low- and middle-income countries (LMICs), where access to care and treatment options are limited. Pneumococcal prophylaxis and pneumococcal vaccination have decreased invasive pneumococcal disease in SCA, but infection remains a common cause of morbidity in African children with SCA. In our recently completed study of hydroxyurea use in SCA, conducted at the Mulago Hospital Sickle Cell Clinic, in Uganda, children with SCA had an average incidence of 0.7 infections/child/year classified as “severe or invasive” infections (NOHARM study) [5]. In addition, two of the three deaths that occurred in the first year of study were attributed to sepsis. The NOHARM data show that infection in children < 5 years of age is a common, serious, and sometimes deadly complication of SCA in Uganda, and supports data from prior studies demonstrating that mortality and infection rates in African children with SCA are highest in children < 5 years of age [6].

Zinc is an essential mineral that plays a critical role in the immune system [7]. It contributes to our first line defense against infectious disease, the skin barrier [8]. Zinc deficiency damages epidermal cells as well as the lining of the gastrointestinal and pulmonary tract [9]. Moreover, zinc deficiency can impair numerous mediators of host immunity, including interference with normal development and function of neutrophils, natural killer cells, T lymphocytes, T helper 1 cytokines, B lymphocytes, and macrophages [9–11]. Zinc is a second messenger of immune cells, an anti-inflammatory agent, and an antioxidant [7]. Free intracellular zinc participates in signaling events [12, 13] and may be used by the innate immune cells as an antimicrobial agent. In animal [14] and human [15] studies, infection is a common complication of zinc deficiency.

There is widespread evidence of zinc deficiency in the context of SCA with studies showing urinary zinc loss in SCA [16] rather than dietary deficiency [17]. This is supported by global data showing reduced zinc levels or zinc deficiency in 15 out of 18 studies in children or adults with SCA, including studies from North America (USA, Canada), South America (Brazil), the Middle East (Saudi Arabia, Turkey, Iraq, Jordan), and Africa (Nigeria, Uganda) [16, 18–34]. Subsequent studies have shown that urinary zinc loss is likely due to impaired renal tubular reabsorption of zinc [35], presumably due to tubular damage from SCA, and that bone degradation, particularly during painful vaso-occlusive crisis (VOC), may lead to increased release of zinc and subsequent loss in the urine, resulting in further depletion of body zinc levels [36]. Thus, zinc may be lost by multiple mechanisms in children and adults with SCA, providing biological plausibility for the findings of decreased zinc levels and frequent zinc deficiency in SCA.

Multiple randomized controlled trials of zinc supplementation for infection prevention have been conducted in LMICs, where infections are the leading causes of morbidity and mortality in children. A review by Yakoob et al. [37] concluded zinc supplementation in children < 5 years of age for at least 3 months reduced the incidence of pneumonia or diarrhea by 19% and 13%, respectively. Further, routine use of zinc supplementation for the clinical management of diarrhea has been approved for use between 10 and 14 days at a dosage of 20 mg/day for children > 6 months of age or 10 mg/day in children < 6 months of age [38, 39]. Thus, zinc appears promising for the reduction of both of these common causes of morbidity in our target population of children with SCA < 5 years of age.

Given the importance of zinc to immunity and an increased likelihood of zinc deficiency in SCA, zinc supplementation could be a way to decrease the risk of infection in SCA. Indeed, studies from the United States and India provide evidence that zinc supplementation may decrease the risk of infection in adults and older children (> 12 years of age) with SCA [40–42]. However, the studies had relatively small numbers, and none were conducted in children < 5 years. Despite these limitations, the findings were strikingly consistent among all studies, with reductions in infection ranging from 47 to 95% with zinc supplementation compared to placebo. Evidence from other studies, particularly in LMICs, suggest that children with SCA may particularly benefit from zinc supplementation to reduce infection and other complications, as summarized in a review article by Dekker et al. [43].

Zinc supplementation is also known to reduce incidences of vaso-occlusion and VOCs in SCA patients, which can result from the sickling phenomenon in abnormal hemoglobin seen in SCA (HbS) [44]. Tissue damage due to VOCs results in complex biochemical, neurologic, and electrochemical events that culminate in pain, which is the number one cause of hospital admissions among patients with SCA [44]. The studies by Bao et al. [40], Prasad et al. [41], and Gupta et al. [42] show a reduction in VOCs, with a reduction of 66% in the study by Bao et al. (not statistically significant) and reductions of from 53 to 58% in the studies by Prasad et al. and Gupta et al. (both statistically significant, *P* <  0.03). Thus, there is evidence that zinc supplementation may be beneficial in reducing the risk of both infections and VOCs in SCA.

Zinc deficiency among children as young as 5 months of age with SCA has also been associated with decreased height and weight, poor muscle mass, and delayed sexual and skeletal maturation [45], which suggests that zinc supplementation may improve growth in the proposed study population of children with SCA < 5 years of age. Studies of zinc supplementation in SCA over 4–12 months have in fact shown height gain and weight gain in adults [16, 46], adolescents [47], and children 4–10 years of age [26, 48]. The association of zinc deficiency in children as young as 5 months of age with decreased height and weight [45] suggests that zinc supplementation may also improve growth in the proposed study population of children with SCA < 5 years of age.

Multiple studies report inadequate zinc intake by Ugandan children [49, 50], with 98% of children younger than 5 years of age estimated to consume less than the recommended nutrient intake (RNI) prescribed by the World Health Organization [49]. The Ugandan diet is rich in phytate, the storage compound of phosphorous in plants, and a potent inhibitor of zinc absorption. The burden of zinc deficiency in Ugandan children is thus assumed high, although the difficulty of measuring a biomarker for zinc in the blood has hindered accurate assessment of the prevalence of zinc deficiency in Uganda and much of the world. Zinc is a common contaminant in the field, making collection of blood into metal-free equipment a prerequisite of accurate assessment [51]. Studies by Dr. Ware and colleagues show that renal dysfunction, in the form of glomerular hyperfiltration, starts in infancy, suggesting that zinc loss in urine likely also starts to occur during this time period, and that young children with SCA in sub-Saharan Africa may be a high-risk group for zinc deficiency as a result of dietary deficiency combined with renal zinc loss [52].

There is evidence that zinc deficiency is common in Ugandan children with SCA [25]. Even in the absence of zinc deficiency, however, there is evidence that zinc supplementation may be beneficial. Fung et al. [26] found that growth in children with SCA improved with zinc treatment even in some children with normal zinc levels, and that zinc levels did not increase in the children supplemented with zinc despite improved growth. This may be because release of free zinc into plasma often occurs during hemolysis, which is constantly present in children with SCA, and so plasma zinc levels may appear normal despite whole body zinc levels being low. Thus, given the high risk of zinc deficiency in this population even with “normal” plasma levels, it is most appropriate to plan a study with supplementation of all children with SCA randomized to zinc supplementation, rather than restricting supplementation to children with low plasma zinc levels. In light of the very strong safety profile of zinc supplementation in children in numerous studies in LMICs (reviewed in [37]), the potential benefits outweigh the minimal risks.

Ugandan children with SCA represent a high-risk group of children under the age of 5 years that are at risk of severe and invasive infections and suffer considerable morbidity associated with VOCs. Based on a growing body of evidence showing that zinc deficiency and zinc loss are common in SCA, that this deficiency may be exacerbated by zinc-deficient and high-phytate diets in Uganda, and that supplementation is effective in reducing invasive bacterial infection in vulnerable groups, we believe a prospective clinical trial evaluating the efficacy of zinc supplementation to reduce infection in children aged < 5 years is warranted as they are most likely to benefit from the intervention. Zinc may also reduce the incidence of VOCs and improve growth, thereby improving the quality of life of children with SCA. Here, we describe the protocol for a randomized, double-blind, placebo-controlled trial evaluating daily zinc supplementation versus placebo for infection prevention in Ugandan children with SCA.

## Methods/design

### Study design

**Fig. 1:**
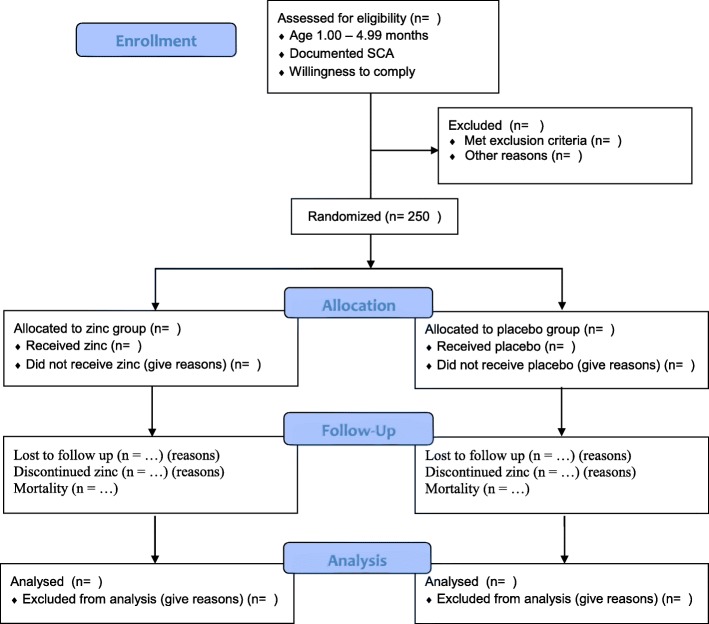
Participant flow diagram. The participant flow diagram illustrates randomization of 250 children with sickle cell anemia to zinc treatment or placebo consistent with the Consolidated Standards of Reporting Trials (CONSORT) 2010 statement

The study is a randomized, double-blind, placebo-controlled clinical trial of oral zinc supplementation (10 mg zinc sulfate) or placebo, administered once daily, to reduce the incidence of severe or invasive infection in Ugandan children with confirmed SCA between 1 and less than 5 years of age. The participant flow diagram is shown in Fig. 1. The study protocol has been developed in accordance with the Standard Protocol Items: Recommendations for Interventional Trials (SPIRIT) guidelines (see Additional file 1 for the SPIRIT checklist).

### Study objectives

The primary objective of the study is to determine if zinc supplementation reduces severe or invasive infections in Ugandan children 1.00–4.99 years of age with SCA. The study will also assess four secondary outcomes that may be affected by zinc supplementation in children: 1) incidence of all clinically diagnosed infections; 2) incidence of culture or PCR-confirmed bacterial infections; 3) incidence of VOCs; and 4) change in height-for-age z-score.

### Eligibility criteria

#### Inclusion criteria

Inclusion criteria include the following:

1. Documented sickle cell anemia (HbSS or HbS/β^0^ thalassemia supported by hemoglobin electrophoresis)
2. Age range of 1.00–4.99 years, inclusive, at the time of enrollment
3. Weight at least 5.0 kg at the time of enrollment
4. Willingness to comply with all study-related treatments, evaluations, and follow-up

#### Exclusion criteria

Exclusion criteria include the following:

1. Known other chronic medical condition (e.g., HIV, malignancy, active clinical tuberculosis)
2. Severe malnutrition determined by impaired growth parameters as defined by WHO (weight-for-length/height or height-for-age z-score < − 3, using WHO growth standards)

### Study setting

Study participants will be recruited from the Nalufenya Sickle Cell Clinic (NSCC) in the Children’s Ward at the Jinja Regional Referral Hospital in Jinja, Uganda. NSCC serves a region of high malaria transmission intensity along the shores of Lake Victoria. The clinic has over 3500 registered SCA children and is run by two pediatricians, assisted by medical officers, nurses, and counselors. Nalufenya Children’s Ward, part of Jinja Regional Referral Hospital, has been the site of several epidemiologic and clinical studies of children [53].

### Treatment groups

Children will be randomly assigned to receive dispersible zinc sulfate tablet (10 mg) or identical placebo tablet in a 1:1 ratio. Zinc tablets will be certified as manufactured under good manufacturing process (GMP). Each tablet is designed to dissolve completely in a small amount of water or breast milk in < 3 min, removing the need for small children to swallow a pill. Caregivers will be instructed to place the pill in 5–10 mL of clean water or breast milk, stir gently, and wait 3 min until the pill is completely dissolved. Both active tablets and placebo will have the same flavoring added to enhance palatability.

### Randomization and blinding

Block randomization will be used for this study. Children will be randomized (in blocks of eight) into treatment groups by order of entry in the study, based on a pre-determined blinded randomization list created and managed by an Indiana University study data manager. Treatment group will be provided to the study pharmacist, who will know whether the child is randomized to group A or B, but will not know which group is zinc or placebo. The study pharmacist will have identical appearing A or B tablets and will provide the appropriate medication to the child. The designation of A or B on packets will be removed by the pharmacist prior to dispensing the study drug so the medication will appear identical to study staff and parents/children. The child’s study identification number will be recorded and treatment group may only be determined by comparing the child’s study id to the blinded list, which only the Indiana University data manager will have access to until the study is completed or stopping rules are reached.

### Outcome measures

The primary outcome to be assessed in the ZIPS trial is the incidence of severe or invasive infections. At all unscheduled sick visits children will be evaluated for clinical evidence of infection by taking a clinical history and exam and diagnostic work up. Children with a measured axillary temperature of ≥ 37.5 °C will have blood obtained for a malaria smear and a blood culture for a measured fever of ≥ 38 °C. Children with history of fever or temperature ≥ 37.5 °C and age-specific tachypnea and cough will have a chest radiograph obtained.

Severe or invasive infections will include abscesses, bacteremia, cellulitis, diarrhea, dysentery, malaria, meningitis/encephalitis, osteomyelitis, pharyngitis/tonsillitis, pneumonia/acute chest syndrome, sepsis, and acute sinusitis using standard definitions (Table 1). Other common infections in this age group (e.g., acute upper respiratory infection (URI), otitis media, conjunctivitis, tinea capitis, tinea corporis) will be recorded and included (along with the severe and invasive infections) in the category of “clinical infections”. Viral infections with a well-defined clinical picture (e.g., measles, varicella) will be defined by clinical signs and symptoms.

**Table 1:** Definitions for severe or invasive infections in ZIPS study

Infection	Definition
Abscess	Opaque, fluid-filled/fluctuant collection on skin (with purulent discharge if drained)
Bacteremia	Children with a positive blood culture with a true pathogen (e.g., *S. aureus*, *S. pneumoniae*, *Salmonella*, other Gram-negative infections)
Cellulitis	Area of reddened, warm skin in a child with a history of fever or measured axillary temperature of ≥ 37.5 °C
Diarrhea	More than three loose stools in a 24-h period
Dysentery	Fever with bloody stools
Malaria	Measured fever (axillary temperature ≥ 37.5 °C) or fever by history and *Plasmodium* species infection on blood smear
Meningitis/encephalitis	Fever with 1) nuchal rigidity or altered mental status and 2) CSF with > 5 WBC or with positive CSF culture for meningitis-associated organisms (e.g., *S. pneumoniae*, *N. meningiditis*, *H. influenzae*)
Osteomyelitis	Fever with bone pain, redness of skin over bone and x-ray findings consistent with osteomyelitis
Pharyngitis/tonsillitis	Inflamed, erythematous pharynx and/or tonsils, with pharyngeal or tonsillar exudates
Pneumonia/acute chest syndrome (ACS)	Clinical syndrome characterized by a new pulmonary infiltrate and at least three of the following: chest pain, temperature greater than 38.5C, tachypnea, wheezing, or cough Children who have three or more of the above symptoms/signs must get a chest x-ray*
Sepsis	Meets modified criteria for SIRS/sepsis in International pediatric sepsis consensus guidelines (two or more of the following criteria, one of which must be abnormal temperature: T ≥ 38.5 °C, age-specific tachycardia, age-specific tachypnea, age-specific leukopenia) Modified to remove leukocytosis because, per NOHARM study data, > 80% of children with SCA at Mulago Hospital will have age-specific leukocytosis at baseline, which is an IPSC criterion for SIRS/sepsis. Since SIRS in a child with SCA is always suspected to be due to infection, we will use the term sepsis
Sinusitis (acute)	Congestion, nasal discharge or cough for more than 10 days without improvement; or symptoms of congestion with purulent nasal discharge for more than 3 days
Urinary tract infection	Symptoms (fever with urinary frequency, burning or new incontinence after prior toilet training) plus urinalysis positive for LE or nitrite OR clean catch urine culture with > 100,000 colonies of a single pathogen

*Any child with a standard clinical diagnosis of pneumonia (clinical signs above) will be treated for pneumonia regardless of CXR findings, as this is Nalufenya Sickle Cell Clinic protocol. Chest x-rays will be read by an on-call radiologist for acute clinical care, and also saved for reading by a second radiologist. Specific criteria will be assessed by both radiologists, and only children who meet criteria from the WHO Radiology Working Group for pneumonia will be given a final diagnosis of pneumonia (Cherian T et al., Bulletin of WHO, 2005;83:353–359). Children who do not meet radiographic criteria will be given a final diagnosis of “respiratory infection” and not included in primary category of “severe or invasive infections” that constitutes the primary study endpoint. They will be considered for the secondary endpoint of “all clinical infections”. *CSF* cerebrospinal fluid, *IPSC* International Pediatric Sepsis Consensus, *LE* leukocyte esterase, *SIRS* systemic inflammatory response syndrome, *WBC* white blood cells

Secondary outcomes of the study include: 1) clinical infection, as described above; 2) culture confirmed bacterial infection (e.g., bacteremia, urinary tract infection, tonsillitis, abscess, osteomyelitis, meningitis) or PCR-confirmed infection with *Chlamydophila pneumoniae* or *Mycoplasma pneumoniae* (from nasopharyngeal swab) in children with pneumonia/acute chest syndrome (pneumonia (acute lower respiratory infection) will be defined as history of fever or measured axillary temperature ≥ 37.5 °C, with age-specific tachypnea, cough, and an infiltrate and/or effusion on chest x-ray consistent with pneumonia); 3) VOC—pain with the requirement for oral morphine or IM diclofenac, per Sickle Cell Clinic (SCC) guidelines; 4) change in height-for-age z-score, from enrollment to 12 months follow-up, calculated using WHO standards.

### Safety

Zinc is approved for use for the treatment of diarrhea, where it has been shown to reduce the duration of diarrheal illness [54]. It is widely available in Uganda and has a well-established safety record. The primary side effect of zinc is vomiting. For children who do have problems with vomiting, parents will be told to give the zinc with food, as this can decrease vomiting. Zinc may interfere with copper absorption [55]. We will measure copper levels from samples at baseline and 12 months to see if copper levels are affected by one-year of zinc supplementation.

Adverse events (AEs) will occur commonly in a trial involving children with SCA, although the majority of events are likely due to the underlying disease process and risk of infections in childhood and not to study medication. AEs will be defined according to Good Clinical Practice (GCP) and will be logged prospectively and tabulated at the end of the study, disaggregated by study arm. They will be defined using the Common Terminology Criteria for Adverse Events (CTCAE) version 4.0 available since 2009 (Tables 2 and 3), where AEs are categorized by organ system and graded by severity. Serious adverse events (SAEs) will adhere to standard definitions (any life-threatening event hospitalization or death); however, since hospitalization is common in children with SCA, we will use hospital stay of more than 7 days to define hospitalization-related SAEs in this study. This is based on the knowledge that children who are admitted with sickle cell-related conditions (such as anemia requiring transfusion, acute chest syndrome, and stroke) have an average length of stay of approximately 7 days. A SAE for this study will therefore include hospitalization for more than 7 days.

**Table 2:** Sickle cell disease symptoms and associated conditions (Common Terminology Criteria for Adverse Events (CTCAE) version 4.0)

Acute chest syndrome	Decreased lung function	Hypertension	Renal papillary necrosis
Adenotonsillar disease	Delayed growth/puberty	Hypocalcemia	Reticulocytopenia
Albuminuria	Depression	Hyposthenuria	Reticulocytosis
Amenorrhea	Dizziness	Nephropathy	Retinopathy
Anemia (severe)	Electrolyte imbalance	Osteomyelitis	Retinal hemorrhage
Aplastic crisis	Elevated urinary urobilinogen	Pain, back	Rhabdomyolysis
Arthralgia	Elevated serum transaminases	Pain, chest	Seizure
Avascular necrosis of hip/shoulder	Elevated transcranial doppler (TCD) ultrasound velocities	Pain, joint	Septicemia
Bacteremia	Fever	Pain, long bone	Silent organ infarction
Bone infarction	Empyema	Pain, severe abdominal	Skin ulcer
Cardiac arrythmia	Hand-foot syndrome/dactylitis	Pain, sternal or rib	Splenic sequestration
Cardiomegaly	Headache	Priapism	Splenomegaly
Cerebrovascular accident	Hematuria	Proteinuria	Stroke
Cholecystitis	Hemiplegia	Pneumonia	Transient ischemic attack (TIA)
Cholelithiasis	Hemolysis	Pulmonary embolism	Transfusion, unanticipated
Cognitive dysfunction	Hepatic sequestration	Pulmonary hypertension	Vaso-occlusive pain
Constipation	Hepatomegaly	Pulmonary infiltrate on chest x-ray	
Cranial nerve palsy	Hospitalization > 24 h	Pyelonephritis	
Death	Hyperbilirubinemia	Renal failure	
Decreased renal function	Hypersplenism	Renal insufficiency

**Table 3:** Laboratory exceptions to the CTCAE list (version 4.0, guidelines)

Parameter	Grade 2	Grade 3	Grade 4
Hemoglobin (Hb) (gm/dL)	5.0–6.0	4.0–4.9	< 4.0
Total WBC (× 10^9^/L)	1.0–1.999	0.5–0.999	< 0.5
ANC (× 10^9^/L)	0.5–0.999	0.2–0.499	< 0.2
Platelets (× 10^9^/L)	50–79	20–49	< 20
Total bilirubin (mg/dL)	5.0–10.0	10.1–20.0	> 20.0
AST (IU/L)	150–300	301–1000	> 1000
ALT (IU/L)	150–300	301–1000	> 1000
Creatinine (mg/dL)	2× baseline serum creatinine and value ≥ 1.0	1.6–2.0	> 2.0
ARC (× 10^9^/L) and Hb < 7.0 gm/dL	50–80	10–49	< 10

*WBC* white blood cells, *ANC* absolute neutrophil count, *AST* aspartate aminotransferase, *ALT* alanine aminotransferase, *ARC* absolute reticulocyte count

In addition to the ethics oversight provided by both Ugandan and North American institutions, an external and independent Data and Safety Monitoring Board (DSMB) has been convened to oversee the trial. The study may be discontinued at any time if the DSMB or study team feels that it is in the best interests of study participants to do so. Stopping rules for SAEs will be developed by the study biostatistician in conjunction with the DSMB. Stopping rules will be created only for SAEs, not efficacy or futility, for this study because there is no perceived benefit to an interim analysis of efficacy/futility in this small cohort with a short treatment duration.

### Sample size and power calculation

We will assess reduction in incidence of severe or invasive infections, with or without culture or PCR confirmation. Sample size is based on incidence of severe or invasive infections, using a baseline rate of 0.71 infections/child/year, derived from data of children from the NOHARM study [5]. Power calculations assume an alpha of 0.025 for a one-sided test or 0.05 for a two-sided test. With an incidence of 0.71 severe or invasive infections/year in the placebo group, a sample size of 250 children (with a 10% loss to follow-up) will have 80% power to detect a decrease of ≥ 40% in severe or invasive infection incidence over the 12-month study period. This decrease is smaller than the 47–88% reduction in clinical infection incidence in adolescents and adults in previous studies [40–42], so the study sample size should allow us to detect the expected effects of zinc on infection incidence.

### Proposed analysis

We hypothesize that the incidence of infection in the zinc supplemented group will be ≥ 40% lower than that in the placebo group. Incidence of clinical infection will be compared using Poisson or negative binomial regression analysis. Other factors that are potentially associated with the risk of infection will be included in the regression models as covariates. Similar analyses will be conducted for incidence of culture or PCR-confirmed bacterial infections, malaria, VOCs, and other sickle-related clinical complications, SAEs, and AEs. Frequency of infection, culture or PCR-confirmed bacterial infection, VOCs, AEs, and SAEs will also be compared using χ^2^ analyses.

### Study assessments

At enrollment, children will have a standard history and physical exam performed. At baseline and 12- month visits, children will receive a complete blood count (CBC) with differential, reticulocyte count, hemoglobin electrophoresis, and plasma stored for zinc testing. Socio-economic status and dietary history will be assessed using a dietary instrument developed and validated for use in a Ugandan population. Urine samples will be collected in zinc-free containers at enrollment and 12-month follow-up in 100 randomly selected children to test for urine zinc levels, to assess degree of urinary zinc loss in study children at baseline and after 12 months of zinc or placebo treatment. Stool samples will also be collected at enrollment and 12-month follow-up from children who are able to provide them, and stored for future microbiome testing.

**Fig. 2:**
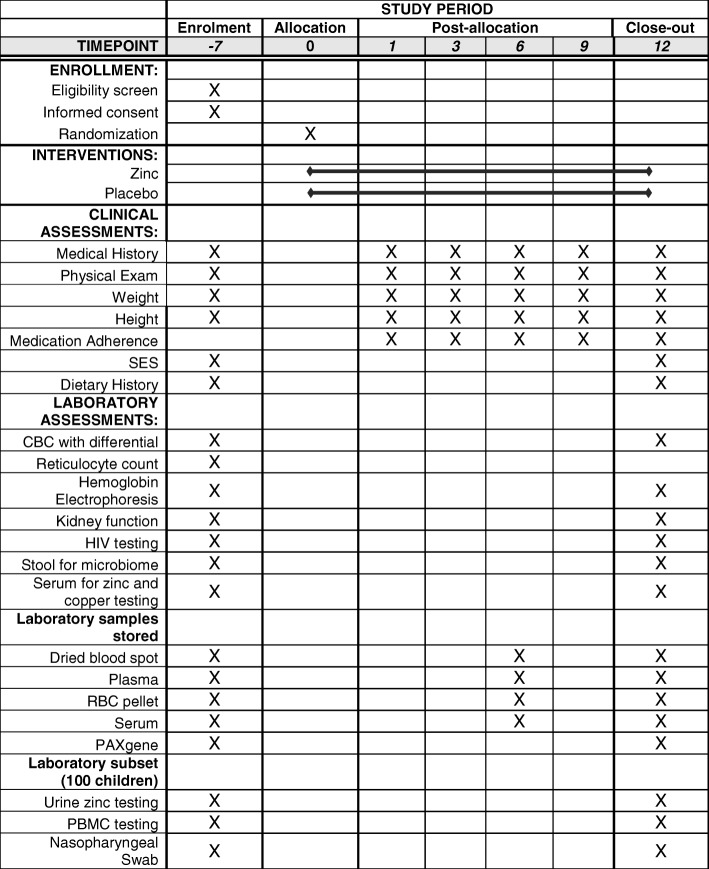
Schedule of enrollment, interventions, and assessments

Children in the study will have scheduled clinic visits at 1, 3, 6, 9, and 12 months to assess study adherence by pill counts, evaluate AEs, assess weight and height, and to refill zinc or placebo tablet supply. Parents or guardians will be asked to bring their children to the NSCC for any illness. Children with illness will be evaluated clinically and be managed according to national and local SCC treatment protocols. Standardized definitions will be provided for the most common infections (Table 1). Children with pneumonia/acute lower respiratory tract infection will have a nasopharyngeal swab collected for later testing by PCR for *Mycoplasma pneumoniae*, *Chlamydophila pneumoniae*, *Bordetella pertussis*, and 17 viral URI pathogens. Children who have clinical signs of pneumonia will receive a chest x-ray. Other labs will be collected as needed for clinical diagnosis, and a blood sample collected and stored for future infectious pathogen or inflammatory response testing. A summary of the schedule of enrollment, interventions, and study assessments is shown in Fig. 2.

### Laboratory testing

Plasma zinc and copper levels and urine zinc levels will be tested in the baseline and 12-month samples at the Wright Lab at Mount Sinai Hospital, New York, NY, or a similarly certified lab for zinc testing. Blood and urine samples will be collected using a tight trace metal-specific sample collection protocol to minimize contamination. Nasopharyngeal swab specimens will be analyzed using the FilmArray® Respiratory Panel (RP; BioFire Diagnostics, Salt Lake City, UT, USA) to test for *C. pneumonaie*, *M. pneumoniae*, *B pertussis*, and 17 viral URI pathogens. Cultures will be done in Bactech bottles (Becton Dickinson, Sparks, MD, USA). Microscopy for *Plasmodium* species by thick and thin smear, with parasite quantification, will be performed as previously described [56].

## Discussion

In this study, if zinc does significantly reduce infection, the next studies would be multi-center studies to determine effectiveness in less well-resourced clinics, likely with a pre-post intervention design, as a large reduction in infection with zinc supplementation in the ZIPS trial might make further placebo-controlled trials ethically questionable. However, if reduction of infection occurs at a more modest magnitude (e.g., a 20–25% reduction, which may not reach statistical significance, but is still important at the population level), then a larger placebo-controlled, multi-center trial would be indicated to obtain sufficient sample size to definitively establish zinc efficacy in children with SCA, and to do so in children from more than one region. Furthermore, zinc supplementation is inexpensive, has minimal side effects, and has been used successfully in children in LMICs in other contexts to prevent infection [37].

Potential limitations and possible alternatives have been considered for this trial. There is a chance that infection rates could be lower in this study than in the NOHARM study [5]. We will analyze infection rates in the cohort as a whole when ~ 50% of the cohort has completed follow-up. If infection rates are lower than expected, we will consider a protocol amendment to increase sample size. This should not increase study costs substantially, unless infection rates are far less than in the prior study. The likelihood of a greatly decreased infection rate is small: we are recruiting from the age group as in our previous study and have data that hospital readmission are more common at NSCC than Mulago (site of NOHARM study); we will be assessing all infections, not a single infection like malaria, which can vary from year to year; and outbreaks of specific infections (e.g., malaria, influenza, measles) that might increase infection incidence were not present in the prior study.

The zinc dose given could be less than that required to prevent infection in children with SCA. However, we believe that the proposed dose (10 mg/day) strikes the best balance between toxicity and efficacy, and has been efficacious in studies of children without SCA, and consequently believe that the best initial study is to look at effects at this dose. A study to compare efficacy of two doses to placebo would require a much larger sample size. Use of plasma zinc levels as a surrogate for efficacy is likely not appropriate, since efficacy for different outcomes has been seen in earlier studies in the absence of a change in levels. A substantial increase in funding would be required for a dose escalation study, as in the absence of a surrogate endpoint it would require a very large sample size. For all of these reasons, we believe that the present study, with a dose of zinc that has been both safe and efficacious in children without SCA, is the best initial study. Study findings should provide data to support or refute the need for additional studies with higher/different zinc dosing.

Zinc supplementation is not standard of care for children with SCA or for otherwise healthy children in Uganda. National guidelines suggest the use of 20 mg of zinc daily during an episode of diarrhea. For children in this study, we will stop study drug treatment (zinc or placebo) if they have an episode of diarrhea, provide zinc (open label) 20 mg during the episode, per national guidelines, and then resume study drug when the diarrhea resolves and they have stopped taking the 20 mg zinc tablets. As noted above, studies suggest that whole body zinc deficiency may be present in children with SCA despite “normal” plasma levels of zinc [26], so the current design of supplementation of all randomized to zinc supplementation is the most appropriate to test the study hypothesis.

Further, current standard of care for infection prevention per the NSCC guidelines for Ugandan children under 5 years of age with SCA includes immunization with pneumococcal 13-valent conjugate vaccine in infancy, pneumococcal polysaccharide vaccination at age 5 years, penicillin prophylaxis to prevent pneumococcal infection, and sulfadoxine-pyrimethamine monthly prophylaxis to prevent malaria. Mebendazole treatment for helminth infections and oral vitamin A supplementation are also recommended for all Ugandan children 1–5 years of age every 6 months. Children also receive daily folic acid to decrease risk of anemia. We will provide any vaccinations, medications, or supplements that a child requires and has not received.

Based on results from our recently completed NOHARM study [5], which showed that short-term fixed-dose hydroxyurea was both safe and effective in the participants of the study, the Ugandan Ministry of Health (MOH) has recommended hydroxyurea for children with SCA with stroke or frequent pain crises. The recent REACH trial further supported the efficacy of hydroxyurea in children with sickle cell disease in African countries including Uganda [57]. Hydroxyurea will be offered to all study participants who the primary clinic physician believes will benefit from the drug. Use of hydroxyurea will not be an exclusion criterion, since there is no good evidence that hydroxyurea therapy affects risk of infections. Instead, hydroxyurea use will be included as a covariate in analyses.

Clinical trial results in adults often differ from those in children, and only a randomized placebo-controlled trial can determine efficacy and the level of efficacy of zinc supplementation for prevention of infection in young children with SCA. Since there are no data on the efficacy of zinc supplementation in children with SCA < 5 years of age, there is clinical equipoise for this trial. Given the evidence of zinc deficiency in children with SCA, the safety and cost-effectiveness of zinc as an intervention, and the efficacy of zinc in prevention of infection in older children and adults with SCA, this randomized controlled trial of zinc supplementation in Ugandan children could lead to an intervention that transforms the health of African children with SCA.

### Trial status

The trial has received all necessary regulatory approvals. The current approved protocol version is 1.0 (version date December 11, 2017). We are currently awaiting receipt of the study drug from the manufacturers. We anticipate a February 15, 2019 start date for recruitment and an August 15, 2020 recruitment completion date.

## Additional file

Additional file 1: Standard Protocol Items: Recommendations for Interventional Trials (SPIRIT) 2013 checklist: recommended items to address in a clinical trial protocol and related documents. (DOC 124 kb)

## Data Availability

The results from this clinical trial have the potential for immediate public health applicability for the sickle cell community around the world. The target audience will be reached through publications, oral presentations, and seminars. Data analysis and manuscript preparation will occur during the last 6–12 months of this proposed trial. All plans for dissemination of study results will be discussed with the investigators and the DSMB before implementation.

## Abbreviations

AE: Adverse event
DSMB: Data safety monitoring board
Hb: Hemoglobin
HbF: Fetal hemoglobin
LMICs: Low- and middle-income countries
NSCC: Nalufenya Sickle Cell Clinic
SAE: Severe adverse events
SCA: Sickle cell anemia
URI: Upper respiratory infection
VOC: Vaso-occlusive crisis
WHO: World Health Organization
ZIPS: Zinc for Infection Prevention in Sickle cell anemia
